# Effects of Sodium-Glucose Cotransporter Type 2 Inhibitors on Residual Kidney Function in Patients Undergoing Incremental Hemodialysis: A Two-Center, Retrospective, Exploratory Cohort Study

**DOI:** 10.3390/medicina62101972

**Published:** 2026-10-12

**Authors:** José C. De La Flor, Elena Jiménez Mayor, Avinash Chandu Nanwani, Celia Rodríguez Tudero, Francisco Valga, Esperanza Moral Berrio, Javier Deira, Jesús Hernández Vaquero, Gabriel de Arriba

**Affiliations:** 1 Department of Nephrology, Hospital Central de la Defensa Gómez Ulla, 28047 Madrid, Spain; jherva5@mde.es; 2 Doctoral Program in Health Sciences, Doctoral School, University of Alcalá (UAH), 28805 Madrid, Spain; 3 Department of Medicine and Medical Specialties, University of Alcalá (UAH), 28805 Madrid, Spain; gabriel.arriba@uah.es; 4 Department of Nephrology, Hospital Santa Bárbara, 42003 Soria, Spain; ejimenezmay@saludcastillayleon.es; 5 Department of Nephrology, Hospital General de Fuerteventura, 35600 Fuerteventura, Spain; achanan@gobiernodecanarias.org; 6 Department of Nephrology, Hospital Nuestra Señora de Sonsoles, 05004 Ávila, Spain; crodrigueztudero@usal.es; 7 Surgery Department, Faculty of Medicine, University of Salamanca, 37007 Salamanca, Spain; 8 Department of Nephrology, Doctor Negrin University Hospital of Gran Canaria, 35010 Las Palmas de Gran Canaria, Spain; evalamae@gobiernodecanarias.org; 9 Department of Medicine, Fernando Pessoa Canarias University, 35450 Las Palmas de Gran Canaria, Spain; 10 Department of Nephrology, Hospital General Universitario de Ciudad Real, 13005 Ciudad Real, Spain; emoral@sescam.jccm.es; 11 Department of Nephrology, San Pedro de Alcantara Hospital, 10001 Cáceres, Spain; javierlorenzo.deira@salud-juntaex.es; 12 Department of Nephrology, Hospital Universitario de Guadalajara, 19002 Guadalajara, Spain

**Keywords:** sodium-glucose cotransporter-2 inhibitors, residual kidney function, kidney replacement therapy, incremental hemodialysis

## Abstract

*Background and Objectives*: The preservation of residual kidney function (RKF) is a major determinant of outcome in patients undergoing incremental hemodialysis (iHD). While sodium-glucose cotransporter-2 (SGLT-2) inhibitors have established nephroprotective benefits in non-dialysis chronic kidney disease (CKD), evidence regarding their role in preserving RKF after the initiation of hemodialysis remains limited. The primary objective of this study was to explore the impact of SGLT-2 inhibitors on the preservation of RKF in incident patients undergoing iHD over a 12-month follow-up. *Materials and Methods*: This study involved a two-center retrospective exploratory observational longitudinal study over 12 months of follow-up, including 32 incident iHD patients (one or two sessions per week, 1WHD or 2WHD). Participants were divided into a study group (SGLT-2iG, n = 15) receiving SGLT-2 inhibitors and a control group (non-SGLT-2iG, n = 17). RKF parameters, including 24 h (h) urine output (UO), urea clearance (KrU), and creatinine clearance (CrCl), were monitored quarterly. *Results*: Over 12 months, 24 h UO increased significantly within the SGLT-2iG (+573.3 mL; *p* < 0.001) and did not change significantly within controls (−202.9 mL, *p* = 0.34). KrU and CrCl showed only non-significant within-group trends (upward in the SGLT-2iG, downward in controls). Between-group differences were not formally tested and are described descriptively. A total of 80% of patients in SGLT-2iG maintained a 1WHD regimen at 12 months, compared with 46.7% in the non-SGLT-2iG. Safety was favorable, with no cases of ketoacidosis or severe infections and a trend toward fewer cardiovascular events in the SGLT-2iG (6.7% vs. 35.3%). *Conclusions*: In this two-center, real-world cohort, SGLT-2 inhibitor use in incident iHD patients was associated with a within-group increase in 24 h UO and non-significant trends toward preservation of KrU and CrCl over 12 months, together with an apparently favorable safety profile. Given the observational design and small sample, these hypothesis-generating findings should be interpreted with caution and warrant confirmation in dedicated randomized controlled trials.

## 1. Introduction

The preservation of residual kidney function (RKF) in patients with end-stage kidney disease (ESKD) undergoing kidney replacement therapy (KRT) is a fundamental clinical objective and a relevant factor influencing survival, quality of life, and a lower incidence of cardiovascular complication [1]. RKF contributes significantly to the control of fluid, blood pressure, anemia, nutrition and electrolyte balance; the elimination of uremic toxins; and the reduction in systemic inflammation [2].

Although no uniform definition exists [3,4], RKF is commonly quantified by residual kidney urea clearance (KrU in mL/min/1.73 m^2^) from a 24 h (h) urine collection [4,5,6,7]. The 2015 National Kidney Foundation (NKF)—Kidney Disease Outcomes Quality Initiative (KDOQI) guidelines recommend measuring kidney urea monthly, which is used to individualize the dialysis dose according to RKF parameters (KrU levels and 24 h urine output (UO)) [3,4,5,6,7,8]. In this context, incremental hemodialysis (iHD) has emerged as an alternative to conventional hemodialysis (cHD), adjusting the dialysis dose to each patient’s RKF, analogously to peritoneal dialysis (PD) [9].

Currently, strategies for preserving RKF on iHD and PD are largely shared and include avoiding nephrotoxic agents and excessive ultrafiltration rates (UFRs), maintaining diuretics and renin–angiotensin–aldosterone system inhibitors (RAASi) whenever possible, careful volume management guided by bioelectrical impedance analysis (BIA) and Point-of-Care Ultrasound (POCUS), protein-controlled diets, use of biocompatible membranes, ultrapure water, a low number of HD sessions, and minimizing intradialytic hypotension (IDH) episodes [10,11,12,13]. Despite these measures, progressive loss of RKF is an almost universal phenomenon, slower in PD and faster in HD, even in incident patients [14,15]. This inexorable decline underscores the need for therapeutic strategies able to preserve or delay it. Therefore, although sodium-glucose cotransporter-2 (SGLT-2) inhibitors have been shown to slow the progression of ESKD and reduce the need for KRT [16,17], understanding the mechanisms by which SGLT-2 inhibitors could preserve RKF in patients undergoing HD is of great clinical and scientific interest [1,18,19].

In recent years, SGLT-2 inhibitors have emerged as a cardio-renal protective drug class that transcends their initial glucose-lowering action. Randomized controlled trials (RCTs) have shown that they slow the progression of chronic kidney disease (CKD) in diabetic and non-diabetic patients by reducing albuminuria and attenuating the decline in estimated glomerular filtration rate (eGFR) [16,17,20,21,22] through reduced glomerular hyperfiltration, a natriuretic effect and restoration of tubulo-glomerular feedback, together with anti-inflammatory, antioxidant and anti-fibrotic properties [19].

However, evidence regarding the impact of SGLT-2 inhibitors on preserving RKF in diabetic and non-diabetic patients undergoing KRT is limited, as these patients were excluded from the pivotal RCTs [1,18,19,23]. Our previous studies of case series in patients undergoing iHD and PD suggest that the use of SGLT-2 inhibitors is safe and associated with reductions in proteinuria, blood pressure, and weight, as well as in preserving RKF [11,24]. Post hoc analyses of RCTs [25,26,27,28,29] and ongoing trials in advanced CKD and dialysis populations, such as the RENAL LIFECYCLE trial [NCT05374291], further support that SGLT-2 inhibitors can be safely used in this setting and may exert favorable effects, including RKF preservation [18,30].

The main objective of our study was to explore the role of SGLT-2 inhibitors on the preservation of RKF in incident patients on iHD compared with non-users over a 12-month follow-up. Rather than comparing dialysis schedules, our aim was to examine whether the nephroprotective and pleiotropic actions of SGLT-2 inhibitors persist below the eGFR thresholds tested in the pivotal trials. Incident patients on iHD with preserved RKF provided an appropriate setting, as RKF is prognostically relevant and routinely quantified through monthly KrU and 24 h UO collections. In this context, SGLT-2 inhibitors could represent an innovative and personalized therapeutic strategy that may help preserve RKF, with the aim of optimizing the management of ESKD and potentially improving prognosis and quality of life.

## 2. Materials and Methods

### 2.1. Study Design and Participants

This is a retrospective observational, exploratory, longitudinal study of two centers based on real-world data; incident patients undergoing iHD through hemodialysis sessions once or twice a week (1WHD or 2WHD) at the Hemodialysis Unit of the Gómez Ulla Central Defense (GUCD) Hospital in Madrid, Spain, who started treatment with any type of SGLT-2 inhibitor (empagliflozin, dapagliflozin or canagliflozin) were classified within the ‘study group’ (SGLT-2iG), while those not receiving SGLT-2 inhibitors from the Chronic Hemodialysis Unit of the San Pedro de Alcántara (SPA) Hospital in Caceres, Spain, were classified as the ‘control group’ (non-SGLT-2iG), with a mean follow-up period of 12 months. The inclusion period spanned from 1 January 2021 to 31 December 2023. Group assignment was determined at the center level according to local prescribing practice, not by individual patient selection. At the study center (GUCD), where SGLT-2 inhibitors are prescribed to virtually all incident iHD patients, we included all consecutive incident iHD patients who met the eligibility criteria and started an SGLT-2 inhibitor within the first three months of KRT (SGLT-2iG). At the control center (SPA), where these drugs are not used, all consecutive eligible incident iHD patients were included (non-SGLT-2iG). Both centers applied identical inclusion and exclusion criteria during the same period; thus, the two-center design, rather than any individual selection, defined the exposed and unexposed cohorts. No individual matching or randomization was performed, assuming baseline comparability based on clinical and temporal criteria. In the SGLT-2iG, treatment was initiated within the first three months following the start of KRT (baseline parameters). All patients who received SGLT-2 inhibitors provided their written informed consent following a comprehensive discussion regarding the known and potential risks and benefits associated with the off-label use of these drugs. The detailed clinical rationale for this practice is meticulously documented in their medical records.

Eligible patients were incident iHD patients who started with 1WHD (KrU > 4 and <7 mL/min/1.73 m^2^ adjusted body surface area (BSA) and 24 h UO > 1000 mL/day) [31] and 2WHD (KrU > 2.5 and <4 mL/min/1.73 m^2^ of adjusted BSA, and 24 h UO > 500 mL/day) [32]. In general, it is advised not to start HD with a KrU > 7 mL/min/1.73 m^2^. During follow-up, dialysis frequency was increased from 1WHD to 2WHD if at least one of the following events occurred: KrU > 2.5 and <4 mL/min/1.73 m^2^ (confirmed in two consecutive monthly reviews), excessive interdialytic weight gain (IDWG) requiring UFR ≥ 13 mL/kg/hour over several sessions, inadequate dialysis adequacy, or clinical events requiring additional sessions (poorly controlled hyperkalemia above 6 mEq/L despite treatment with potassium binders or poorly controlled hyperphosphatemia above 5.5 mEq/L despite treatment with phosphorus binders). Patients on the 2WHD schedule were transferred to 3WHD if their KrU was <2.5 mL/min/1.73 m^2^ or affirmed at least one event already mentioned previously. Both centers prescribed escalating iHD to a 2WHD or 3WHD schedule, together with biochemical and diagnostic monitoring applied to both study groups derived from the A Randomized Controlled Trial to Assess the Safety and Efficacy of Incremental Hemodialysis in Incident Patients trial (IHDIP) design [31]. Nevertheless, the standardized volume-management strategy used to adjust dry weight, which combines BIA with POCUS (Lung Ultrasound (LUS) and Venous Excess Ultrasound Score (VExUS)), was implemented exclusively at the study center (GUCD Hospital), whereas the control center (SPA Hospital) did not adopt this follow-up approach.

On the other hand, patients under 18 years of age; those who were unable to understand or did not sign the informed consent form; those unable or unwilling to collect 24 h urine samples monthly; those with a UO ≤ 200 mL/day or anuria; those who had a history of failed kidney transplant; those transferred from PD due to failure of the technique; patients with urgent or unscheduled HD initiation; patients with a history of type 1 diabetes mellitus (T1DM); patients with active urinary tract infections (UTIs) at the time of initiating SGLT-2 inhibitor treatment, active neoplasia or a life expectancy of less than 12 months; and those with cardiorenal syndrome, ischemic heart disease or hemodynamic decompensation with admission within 3 months prior to inclusion within this study were excluded.

The primary endpoint of our study was to evaluate the change in RKF parameters (KrU, residual renal creatinine clearance (CrCl), and 24 h UO) from baseline to 12 months in incident patients undergoing iHD (1WHD or 2WHD) treated with SGLT-2 inhibitors, with a non-user group as a descriptive reference. Secondary endpoints comprised the within-group change from baseline to 12 months in the following parameters: dry body weight, blood pressure, 24 h urine protein, hemoglobin and ferrokinetics; serum electrolytes and 24 h urinary excretion of sodium (Na^+^), potassium (K^+^), chloride (Cl^−^), calcium (Ca^+2^), phosphorus (P), magnesium (Mg^+2^), and uric acid (UA) levels; and body composition and hydration assessed by BIA and nutritional ultrasound (NUS) of muscle and preperitoneal visceral fat (PPVF). The safety of SGLT-2 inhibitors for iHD patients was also a key secondary outcome, specifically monitoring adverse events (AEs) such as genital infection, urinary tract infection (UTI), catheter-related infection, cardiovascular events (CVEs) (acute myocardial infarction disease (AMI), heart failure (HF), peripheral vascular disease (PVD), stroke), hypoglycemia, euglycemic diabetic ketoacidosis (EDKA), hypersensitivity, amputation, hospitalization (any cause), cardiovascular death, death (non-cardiovascular cause), and intradialytic hypotension (IDH) (defined as a decrease in systolic blood pressure (SBP) ≥ 20 mmHg), in combination with associated clinical and nursing intervention [33], calculated as the rate of IDH episodes per session (total number of IDH episodes/total number of sessions).

### 2.2. Ethics

Each participant provided written informed consent for the utilization of data from their medical records. The study protocols were performed in strict compliance with the Declaration of Helsinki, following ethical approval granted by the Clinical Research Ethics Committee of Hospital GUCD (Approval code number: 63/25).

### 2.3. Data Collection

Baseline comorbidities and medications were obtained within three months before inclusion. Baseline demographic data and anthropometric variables included age, sex, weight in kilograms (kg), height in meters and body mass index (BMI) in kg/m^2^. Also, malnutrition risk parameters were collected using the malnutrition inflammation score (MIS), malnutrition screening tool (MST) [34], and seven-point subjective global assessment (7p-SGA) scale [35]. Malnutrition was diagnosed using the protein-energy wasting method of the International Society for Renal Nutrition and Metabolism (PEW-ISRNM-2014), as well as sarcopenia risk with the SARC-F scale, and the diagnosis of sarcopenia was made according to the 2019 European Working Group on Sarcopenia in Older People (EWGSOP2) consensus criteria [36].

The parameters for assessing RKF as 24 h UO (mL/24 h), KrU (mL/min/1.73 m^2^) and residual kidney CrCl (mL/min/1.73 m^2^) were obtained quarterly from baseline to 12 months after the start of iHD. KrU was calculated using the standard method [37], with the following formula: KrU (mL/min) = U_urea_ × (U_vol_/1440 min)/P_urea_. U_urea_ and P_urea_ are the urine and plasma concentrations of urea, respectively. U_vol_ is the UO in 24 h. Urine collection was obtained from the last 24 h of the interdialytic interval ending before the next HD session, and P_urea_ was expressed as the average of post- and pre-dialysis urea concentration between the two consecutive HD sessions. In addition, in patients who already started KRT, KrU was calculated using urea kinetic modeling (UKM) formulae with SPEEDY (SPEEDY-1 version, Acquaviva delle Fonti, Italy) software [38] and Solute Solver software (Solute Solver-1 version, Chicago, IL, USA) [39]. Data on proteinuria measured in grams over 24 h were also collected, and the urine protein-to-creatinine ratio (uPCR, mg/g) and urine albumin-to-creatinine ratio (uACR, mg/g) were measured in a first-morning urine sample. In addition, serum analytical parameters and data on 24 h urinary excretion of Ca^+2^, P, Mg^+2^, Na^+^, K^+^, Cl^−^ and UA were also collected at 0, 3, 6, 9, and 12 months.

The analysis of body composition and hydration was assessed at 0, 6 and 12 months by segmental multifrequency BIA InBody S10 (InBody Japan Inc., Tokyo, Japan), using identical instrumentation and schedule at both centers. In addition, NUS parameters were measured in both centers, following the protocol by García-Almeida et al. [40]. Handgrip (HGS, kg) was evaluated by dynamometry with the CAMRY dynamometer model EH101 (Zhongshan Camry Electronic Co., Ltd., Zhongshan, China). The arm circumference (AC, cm) and waist circumference (WC, cm) were also collected. Triceps and suprailiac skinfold thicknesses were measured following the procedures established by the International Society for the Advancement of Kinanthropometry (ISAK) protocol [41], using a Harpenden skinfold caliper (HSK-BI, British Indicators, Chichester, UK). The NUS, HGS, AC, WC, triceps and suprailiac skinfold thicknesses were assessed at 0 and 12 months; intermediate 6-month values for these specific measures were not systematically available. Both centers have experience in the early detection of malnutrition and sarcopenia using BIA and NUS, and both currently operate structured management programs that combine intradialytic exercise and oral protein supplementation. However, during the study period (2021–2023), these structured programs had not yet been implemented, and none of the patients enrolled at either center participated in them. Patients received clinical nephrology care in real-world practice, which in some cases included oral nutritional supplementation; these interventions were not systematically recorded and could not be analyzed.

Ultimately, information regarding efficacy and hemodialysis parameters (dialysis dose with total urea clearance (Kt), blood flow rate (QB), and arterial/venous pump flow (APF/VPF)), were collected quarterly during the follow-up period.

In summary, both centers shared the same dialysis framework, the same modality (post-dilution online hemodiafiltration (OL-HDF) with biocompatible polysulfone dialyzers) and identical BIA and NUS instrumentation and schedule, and differed only in SGLT-2 inhibitor exposure and in the multiparametric dry-weight protocol (BIA plus POCUS), which was applied only at the study center.

### 2.4. Statistical Methods

Continuous variables were presented as means and standard deviations (SD) or as medians and interquartile ranges (IQR) depending on the normality of the sample. Categorical variables were displayed as frequencies and valid percentages. The Kolmogorov–Smirnov test was used to determine the normality of the data distribution and the applicability conditions for parametric tests. Given the observational, non-randomized two-center design, in which treatment was assigned at the center level and the two cohorts differed at baseline in RKF, formal between-group comparisons would have been confounded by center and by indication. Moreover, the small sample size (n = 32) provided insufficient statistical power for reliable between-group inference and would yield unstable estimates. We therefore restricted the formal analysis to the within-group longitudinal trajectory of each outcome, in which each patient serves as their own control, and which is inherently more robust to baseline imbalance. For each group, the change in KrU, CrCl and 24 h UO during follow-up was quantified by the within-patient slope (individual linear regression) and by the within-group change from baseline to 12 months (paired *t*-test or Wilcoxon signed-rank, depending on distribution). Between-group differences were not formally tested and are described descriptively in the text. Safety outcomes were low-frequency, categorical events; given the small number of events, they are reported descriptively (counts and percentages) and were not formally compared between groups. For statistical analysis, STATA 14.0 statistical software (Stata Statistical Software: Release 14. College Station, TX, USA: Stata Corp LP) was used, with a *p*-value < 0.05 considered statistically significant.

## 3. Results

**Figure 1:**
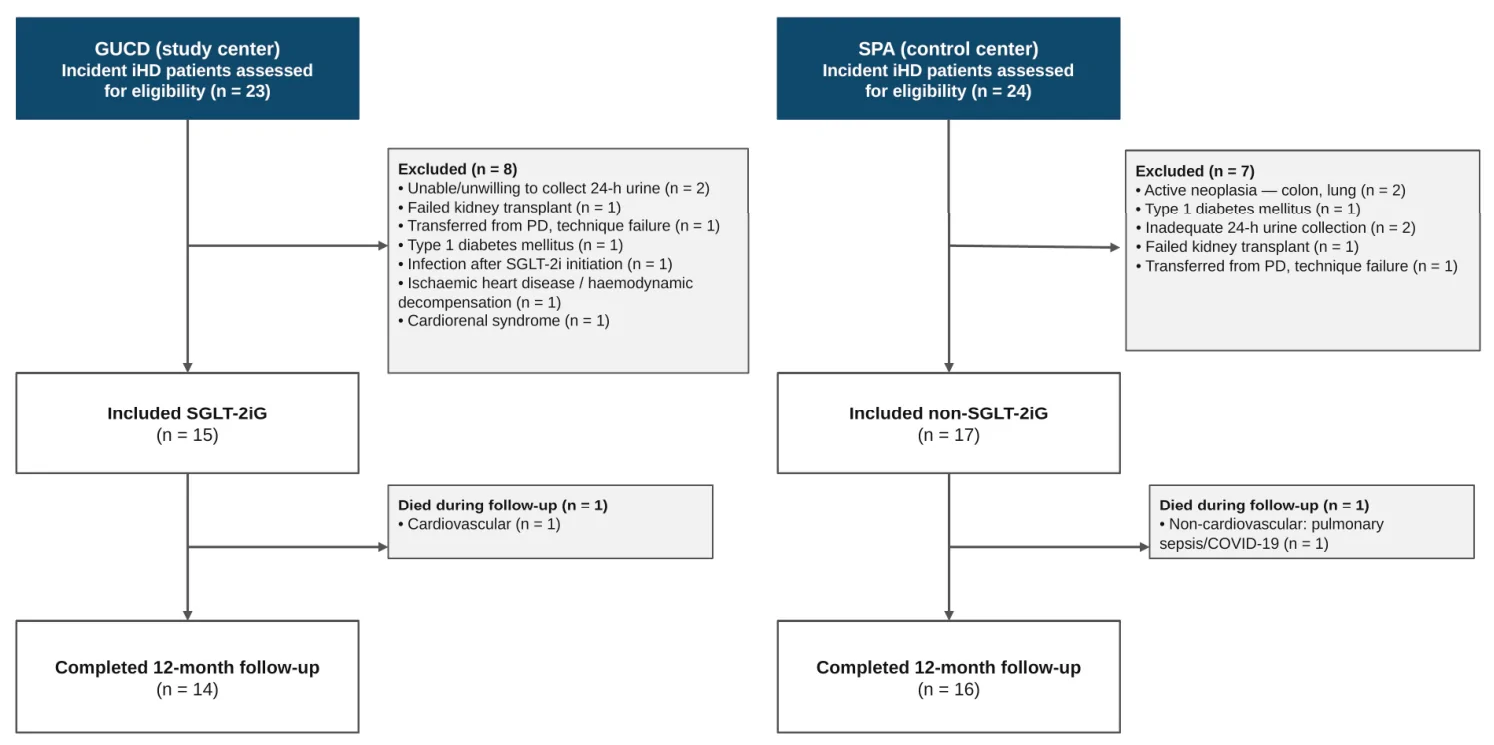
Flowchart of patient inclusion and exclusion criteria. iHD: Incremental hemodialysis; SGLT-2iG: sodium-glucose cotransporter-2 inhibitor group; GUCD: Gomez Ulla Central Defensa Hospital; SPA: San Pedro de Alcántara Hospital; PD: peritoneal dialysis; n: number of patients.

During the study period, 23 and 24 incident iHD patients were assessed for eligibility at GUCD and SPA Hospital, respectively. Eight patients were excluded at GUCD and seven at SPA Hospital (reasons detailed in Figure 1); a total of 32 patients were included. There was a total of 15 patients in the study group (SGLT-2iG) and 17 in the control group (non-SGLT-2iG). The proportion of screened patients excluded was comparable between the centers (8/23, 34.8% at GUCD vs. 7/24, 29.2% at SPA Hospital). During the 12-month follow-up, one patient in each group died and was not replaced; 14 SGLT-2iG and 16 non-SGLT-2iG patients completed follow-up and were included in the 12-month analyses. The participant flow is summarized in Figure 1.

A total of 68.8% of the entire cohort comprised men, with a mean age of 69.9 years (SD = 11.2). Regarding the etiology of CKD, considerable heterogeneity was observed, with diabetic kidney disease (DKD) and chronic tubulo-interstitial nephropathy (CTIN) being the most common. The Charlson comorbidity index was 6.7 (SD = 2). A total of 96.9% had hypertension (HTN). All 100% of patients in both groups were taking loop diuretics, with an average dose of 40 mg of furosemide; the two groups differed only in the addition of an SGLT-2 inhibitor. This study therefore assessed the incremental effect of adding an SGLT-2 inhibitor to a common loop-diuretic background, not SGLT-2 inhibition in isolation. ACEi (angiotensin converting enzyme inhibitors) and ARB (angiotensin II receptor blockers) were used by 100% of the SGLT-2iG and 94.1% of the control group. No patients in either group received either MRA (mineralocorticoid receptor antagonist) or ARNI (Angiotensin Receptor Neprilysin Inhibitor). Type 2 diabetes mellitus (T2DM) was present in more than half of the patients (n = 18, 56.3%), while obesity affected 37.5% (n = 12, 37.5%). The rest of the baseline characteristics of the entire cohort, both this study and the control group, are shown in Table 1.

In the non-SGLT-2iG, all (17) patients started with the 1WHD regimen. The proportion of patients maintaining this regimen progressively decreased to 82.4% at 3 months, 64.7% at 6 months, 53.3% at 9 months, and 46.7% at 12 months in non-SGLT-2iG. In contrast, 86.7% of patients in the SGLT-2iG (13/15) started with the 1WHD regimen. The proportion of patients requiring no more than 1WHD was 100% at 3 months, 86.7% at both 6 and 9 months, and 80.0% (12/15) at 12 months. The type of HD performed in both groups was post-dilution OL-HDF with highly permeable and biocompatible membranes (polysulfone dialyzers). Most had a native arteriovenous fistula (nAVF) (71.9%; n = 23) and the rest had a central venous catheter (CVC) (28.1%; n = 9). After analyzing all HD sessions in each group over the 12-month follow-up period (852 HD sessions in the SGLT-2iG and 900 in the non-SGLT-2iG), IDWG (1.47 ± 1.30 kg vs. 1.55 ± 1.35 kg) and UFR per session (5.03 ± 3.84 mL/kg/h vs. 5.35 ± 4.42 mL/kg/h) were comparable between the two groups. At the start of KRT, RKF was higher in the control group, significantly so for 24 h UO (*p* = 0.009) and KrU (*p* = 0.02), with similar CrCl (*p* = 0.94). The rest of the results are shown in Table 1.

At baseline, the two cohorts differed in several HD-related parameters. Kt (53.3 ± 6.5 L in the SGLT-2iG vs. 45.5 ± 1.4 L in the non-SGLT-2iG), VPF (157.2 ± 9.9 mL/min vs. 190.6 ± 14.0 mL/min), and APF (−161.1 ± 36.7 mL/min vs. −196.1 ± 10.9 mL/min) differed between groups, as SBP and diastolic blood pressure (DBP) were both higher in the SGLT-2iG (157.1 ± 13.9 vs. 143.7 ± 17.5 mmHg; and 82.1 ± 8.1 vs. 73.3 ± 14.7 mmHg). These baseline imbalances reflect the non-randomized, two-center design and are considered in the interpretation of the results. Of note, Kt, APF and VPF are markers of dialysis efficiency and vascular-access blood flow, without an established direct effect on RKF; as both groups had a comparable proportion of AVF and CVC, these differences reflect the technical variability inherent to a two-center, real-world setting rather than a different access type, and are reported descriptively only. Nutritional and functional risk profiles were broadly similar between groups, except for MST; notably, the SGLT-2iG contained no patients with an MST score above 2. The remaining results are shown in Table 1.

### 3.1. Primary Endpoint

**Figure 2:**
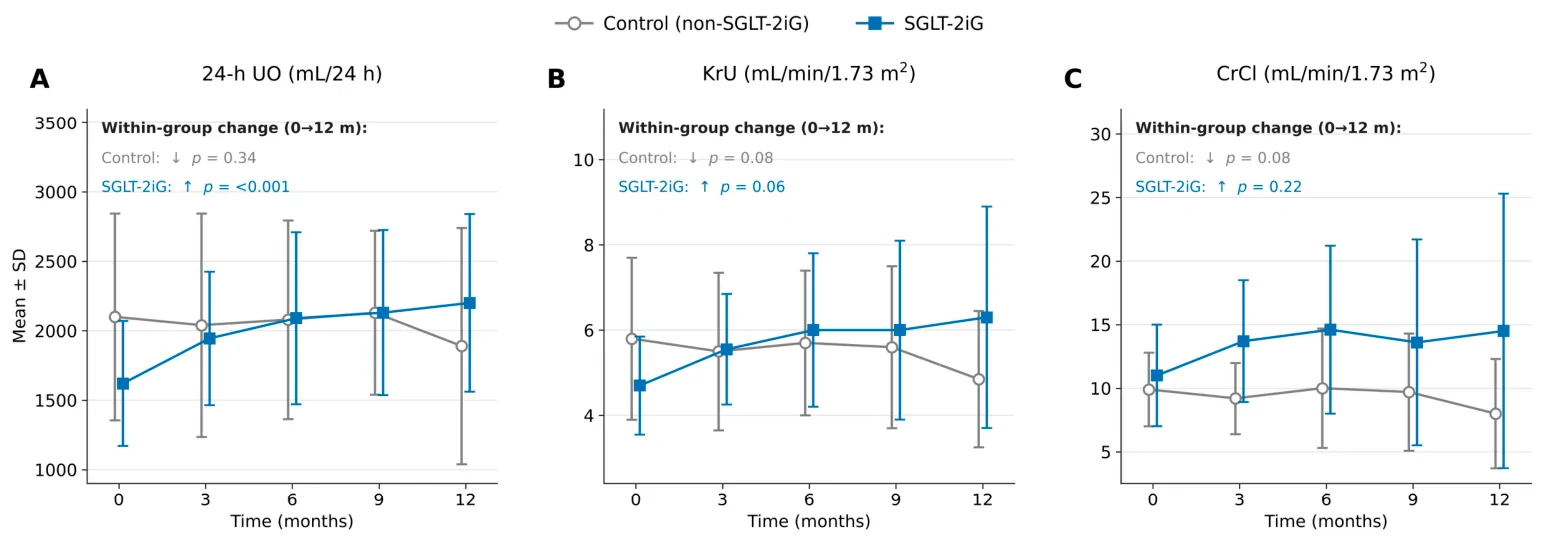
Within-group trajectories of residual kidney function (RKF) parameters during the 12-month follow-up. (**A**) The 24 h urine output (UO); (**B**) residual urea clearance (KrU); and (**C**) residual creatinine clearance (CrCl). Data are shown as mean ± standard deviation at 0, 3, 6, 9 and 12 months for the control (non-SGLT-2iG, gray) and study (SGLT-2iG, blue) groups. The within-group change from baseline to 12 months (paired *t*-test or Wilcoxon signed-rank) is annotated for each group; arrows denote the direction of change. Between-group differences were not formally tested and are described descriptively in the text. Baseline corresponds to the initiation of the SGLT-2 inhibitor (within the first three months of KRT); the equivalent time point was used for the control group.

Regarding the RKF parameters assessed quarterly in both groups from baseline to 12 months (Table 2), within-group trajectories diverged between the two groups. KrU decreased in the controls (5.8 to 4.9 mL/min/1.73 m^2^, *p* = 0.08) and increased in the SGLT-2iG (4.7 to 6.3 mL/min/1.73 m^2^, *p* = 0.06); although neither within-group change reached statistical significance, the two trajectories moved in opposite directions, so that the group with higher values at baseline (controls) was lower at 12 months, a descriptive observation only, as between-group differences were not formally tested. A similar pattern was observed for 24 h UO, which increased within the SGLT-2iG (1620 to 2193 mL/24 h; *p* < 0.001) while remaining essentially unchanged in the controls (2092.9 to 1890 mL/24 h; *p* = 0.34), and for CrCl, which rose within the SGLT-2iG (11.0 to 14.5 mL/min/1.73 m^2^) and declined in the controls (9.9 to 8.0 mL/min/1.73 m^2^). The trajectories of all three parameters are shown in Figure 2.

### 3.2. Secondary Endpoint

Serum laboratory parameters, proteinuria, and 24 h urinary excretion electrolytes were comparable between the two groups at 0, 6 and 12 months (Table S1). Within the SGLT-2iG, hemoglobin increased over the 12-month follow-up (by 2.0 g/dl; *p* = 0.001), whereas within the non-SGLT-2iG, the increase was smaller (0.85 g/dl; *p* = 0.07). This within-group change cannot be attributed to SGLT-2 inhibition, as between-center differences in anemia management, such as hemoglobin targets or erythropoiesis-stimulating agent (ESA) dosing, were not recorded and cannot be excluded. Within the SGLT-2iG, serum beta-2 microglobulin (B2M), UA, low-density lipoprotein (LDL), non-high-density lipoprotein cholesterol (non-HDL-c) and parathyroid hormone (PTH) decreased, and serum bicarbonate increased over the 12-month follow-up. Regarding urinary parameters, the SGLT-2iG showed higher glucosuria and higher urinary excretion of chloride, urea, calcium, sodium and potassium, together with a reduction in proteinuria, uACR and uPCR; these urinary differences between groups are described descriptively (Table S1).

Within the SGLT-2iG, body weight fell (75.7 to 70.8 kg; *p* < 0.001), with a parallel BMI decrease (27.8 to 26.0 kg/m^2^; *p* < 0.001), whereas weight was stable in the controls (78.0 to 79.1 kg; *p* = 0.21) (Table S2). By BIA, the SGLT-2iG showed reductions in total body water (TBW) (38.3 to 34.8 L; *p* = 0.02) and intracellular water (ICW) (22.7 to 21.0 L; *p* = 0.02), as well as body fat mass (BFM) (25.2 to 23.1 kg; *p* = 0.02), with improvements in phase angle (PhA) (*p* = 0.006) and unchanged extracellular water (ECW), lean body mass (LBM), visceral fat, body cell mass (BCM) and basal metabolic rate (BMTR). The modest decrease in fat-free mass (FFM) (50.7 to 47.8 kg; *p* = 0.01) likely reflects fluid loss rather than muscle wasting, consistent with stable appendicular skeletal muscle mass index (ASMI) (8.1 to 8.0 kg/m^2^; non-significant) and improved NUS muscle parameters. Full values are shown in Table S2.

### 3.3. Safety Outcomes

Over the 12-month follow-up, the SGLT-2iG exhibited a favorable safety and tolerability profile. No episodes of EDKA, limb amputation, UTI, genital infection, or hypersensitivity reaction occurred in either cohort. Hypoglycemia was recorded in three (17.6%) patients of the non-SGLT-2iG, and none of the SGLT-2iG. CVEs were less frequent in the SGLT-2iG (6.7% vs. 35.3%). COVID-19 infections occurred in two (13.3%) patients in the SGLT-2iG and three (17.6%) controls, and catheter-related infections in two (13.3%) and three (17.6%), respectively, with a similar distribution between groups. Hospitalizations were rare and balanced, predominantly attributable to COVID-19; other causes of hospitalizations are described in Table 3. Two deaths occurred during follow-up, one in each group: one cardiovascular in the SGLT-2iG and one non-cardiovascular (pulmonary sepsis–COVID-19) in the control arm. The rate of IDH episodes/total number of HD sessions was numerically lower in the SGLT-2iG (descriptive only) (SGLT-2iG 10/852, 1.17%; non-SGLT-2iG 16/900, 1.78%). In the 12 months following the end of this study, three patients in the non-SGLT-2iG group and two in the SGLT-2iG group underwent a kidney transplant (KT) (Table 3).

## 4. Discussion

Our study is the first to use real-world data to assess the role of SGLT-2 inhibitors in preserving RKF in incident patients undergoing iHD, providing preliminary, hypothesis-generating evidence that these agents may help preserve RKF over 12 months, with a non-user group as a descriptive reference. First, our primary outcome findings show that over 12 months, the three RKF parameters (24 h UO, KrU, and CrCL) were preserved or improved within the SGLT-2iG, whereas they declined within the control group, so that the two trajectories moved in opposite directions and reversed the baseline difference between groups. This pattern is consistent with a lower likelihood of a clinically relevant decline in KrU, a factor linked to survival and quality of life in this population, although given the observational design, this between-group difference is described descriptively rather than as a proven effect. Also, 80% of patients in the SGLT-2iG maintained their dialysis regimen of 1WHD, while this percentage in the control group was 46.7% and these patients required a significant increase in session frequency (1WHD to 2WHD, 13.3% patients). Second, patients in the SGLT-2iG showed increased urinary excretion of sodium, potassium, chloride, urea, calcium and UA, along with a reduction in proteinuria. Furthermore, body-composition assessment by BIA within the SGLT-2iG showed that weight loss occurred mainly at the expense of adipose tissue and ECW, without an accompanying reduction in muscle mass. At the 12-month follow-up, NUS parameters (*Y*-axis, cross-section muscle area of the rectus femoris (CS-MARF), muscle area rectus femoris index adjusted by height (MARFI_h_), and PPVF) were also preserved or improved within the SGLT-2iG. These body-composition and muscle findings are descriptive and should not be attributed to SGLT-2 inhibition. No serious adverse events such as EDKA, UTI or genital infections were reported, and a trend toward fewer CVEs was observed in the SGLT-2G (6.7% vs. 35.3%).

Traditionally, there has been reluctance to use SGLT-2 inhibitors with very low eGFR, owing to reduced glucose-lowering efficacy and concerns about the initial dip in glomerular filtration. However, their nephroprotective actions are largely independent of glycemic control: they attenuate glomerular hyperfiltration and reduce proteinuria by restoring tubuloglomerular feedback [42] and exert pleiotropic anti-inflammatory, antioxidant and renal–metabolic effects that mitigate renal hypoxia [43]. Despite an eGFR below 15 mL/min, in our cohort of patients with ESKD undergoing iHD, the preservation of RKF parameters (24 h UO, KrU and CrCl) is a primary therapeutic objective and is of vital importance. Our findings are consistent with the hypothesis that the nephroprotective effect of SGLT-2 inhibitors may persist even in this population, whereby the remaining functional nephrons could respond to SGLT-2 inhibition, reducing hyperfiltration and potentially attenuating the progressive loss of RKF [1,18,19]. Our findings are consistent with the results of a prospective intervention study by Abdelaziz M. et al. [44], involving 40 patients on maintenance HD who were treated with 10 mg of empagliflozin for 12 weeks in the absence of a control group, in which a numerically non-significant improvement in eGFR was observed, accompanied by the preservation of 24 h UO.

Regarding the impact of volume management as a strategy for preserving RKF in HD patients, within the SGLT-2iG, the 24 h UO increased over the 12-month follow-up (from 1620 to 2193 mL/24 h; +573.3 mL; *p* < 0.001). SGLT-2 inhibitors induce a natriuretic effect, thereby generating osmotic diuresis and reducing intraglomerular pressure [23,28,45]. In ESKD, this mechanism persists, continuing to contribute to nephroprotection by restoring tubulo-glomerular feedback [23,45]. In our study, both groups continued their diuretic treatment after starting iHD. Importantly, loop-diuretic use and dose were comparable in both groups (100%; 40 mg/day furosemide), so the divergent RKF trajectories are unlikely to be explained by the diuretic and are more plausibly attributable to the added SGLT-2 inhibition. Therefore, the use of loop diuretics in combination with SGLT-2 inhibitors reduces IDWG, facilitates the management of volume overload, and allows for a more conservative approach to the UFR during dialysis. This is crucial, as high UFRs are a known risk factor for accelerated loss of RKF due to episodes of IDH and recurrent renal ischemia [2], especially in the first few months after HD initiation [13,46,47]. We observed a lower rate of episodes of IDH per total HD sessions in the SGLT-2iG; this may be because, in our hospital (GUCD—study group), we use a standardized protocol to adjust dry weight, employing a multiparameter assessment that includes physical examination, POCUS (LUS, VExUS) and BIA, whereby dry weight is considered a relative value rather than an absolute value. We acknowledge, however, that this multiparametric protocol was applied only at the study center; nevertheless, the fluid-removal burden (IDWG and UFR per session) was comparable between groups, and the baseline differences in dialysis-efficiency and vascular-access flow parameters (Kt, APF, VPF) have no established effect on RKF, so these center-level differences typical of a real-world, two-center study do not readily explain the observed RKF preservation. Unlike our study, the BISTRO (Bio-impedance Spectroscopy Added to a Fluid Management Protocol) study [48,49] RCT, involving 439 incident HD patients across 34 United Kingdom centers, failed to demonstrate that incorporating BIA into a standardized fluid management protocol improves the preservation of RKF compared with standard clinical judgment. This is likely because they only incorporated BIA, unlike our protocol, which went further by incorporating US parameters.

Another important consideration is that the apparent within-group increase in KrU and CrCl should not be interpreted as a recovery of renal function. It is more plausibly explained by the preservation of RKF combined with hemodynamic factors, as correction of chronic fluid overload and venous congestion, together with the natriuretic action of SGLT-2 inhibitors on a loop-diuretic background, may improve renal perfusion and increase measured clearances without an actual change in nephron mass. Furthermore, KrU and CrCl obtained from timed urine collections are influenced by volume status and the completeness of the collection, and CrCl overestimates GFR, owing to tubular secretion—caveats that further support a cautious, descriptive interpretation.

The between-group divergence in RKF parameters was most pronounced during the first six months, with relative stabilization thereafter. This temporal pattern is consistent with the early onset of the natriuretic, osmotic–diuretic and antiproteinuric actions of SGLT-2 inhibitors and with the restoration of tubuloglomerular feedback, which would be expected to have their greatest effect on RKF shortly after initiation, precisely the period in which the decline of RKF after HD initiation is steepest. Part of the later divergence, however, was driven by a steeper decline in urine output and residual clearances within the control group during the final three months; given the two-center, real-world design, unrecorded center-level factors cannot be excluded as contributors to this late trajectory, and the temporal pattern is therefore interpreted with caution.

On the other hand, proteinuria is known to be an independent predictor of worsening and/or loss of RKF in patients with ESKD, including those on dialysis; however, the evidence supporting the latter is limited [46]. Sanchez-Alamo B. et al. [50] recently published a prospective study of incident patients on iHD with OL-HDF in the 2WHD regimen and preserved RKF, which examined how proteinuria levels at the start of HD influence the preservation of RKF. Specifically, patients who started on iHD with nephrotic-range proteinuria (≥3.5 g/24 h) have twice the risk of losing their RKF and progressing to anuria (HR 2.04; 95% confidence interval 1.03–4.02; *p* = 0.04) during their first year of dialysis compared with those with lower levels, a finding that remained consistent across adjusted models. It is important and necessary to implement personalized therapeutic strategies aimed at reducing proteinuria as a means of preserving RKF and improving survival rates in this modality of HD. Interestingly, these researchers found that medications known for their effects in reducing proteinuria and slowing the progression of CKD, such as ACEis and ARBs, did not significantly slow RKF decline. However, their study did not evaluate the additional impact of SGLT-2 inhibitors on reducing proteinuria. SGLT-2 inhibitors have been shown to reduce proteinuria, even in patients with ESKD [1,11,51]. In our study, RAASi use was comparably high in both groups (100% of the SGLT-2iG vs. 94.1% of controls). Within the SGLT-2iG, 24 h proteinuria, uACR and uPCR decreased over the 12-month follow-up (*p* = 0.01, *p* = 0.02 and *p* = 0.01, respectively), whereas within the control group, proteinuria increased. Because RAASi exposure was similar in both groups, this divergent trajectory is unlikely to be explained by differential RAASi use and is consistent with an additional antiproteinuric effect of SGLT-2 inhibition, in line with the findings of the study of Sanchez-Alamo B. et al. [50], who reported no reduction in proteinuria with ACEi or ARB alone. This is also in keeping with our retrospective series of seven diabetic patients on iHD, in which the SGLT-2 inhibitors used were associated with the preservation of RKF (from 4.9 to 7.3 mL/min at 12 months, *p* = 0.028) and a reduction in 24 h proteinuria and uACR by almost 60% without serious adverse effects [11]. Very similar findings were observed in the study by Abdelaziz M. et al. [44]; these authors reported a reduction in the uACR −80 mg/g (*p* = 0.022), along with an improvement in DBP and a reduction in inflammation, in a cohort of 40 diabetic and non-diabetic patients.

Consistent with the known pleiotropic effects of SGLT-2 inhibitors on CKD–mineral and bone disorders (CKD-MBD), we observed a transient, early increase in serum phosphate during the first 6 months and a mild increase in serum magnesium at 12 months within the SGLT-2iG, without consistent or significant changes overall [52,53,54,55,56]. These minor biochemical shifts are in agreement with the class effects described in earlier-stage CKD and were not associated with clinical consequences in our cohort. As these were secondary, non-pre-specified observations in two non-comparable cohorts, they are reported descriptively and are not interpreted further.

In our cohort, within the SGLT-2iG, serum UA decreased at 12 months while urinary UA excretion and clearance increased, consistent with the known uricosuric effect of this drug class [57,58,59], which large cohorts and meta-analyses have linked to reductions in new-onset gout and hyperuricemia, including in CKD [60,61,62,63]. On the other hand, serum sodium, potassium and chloride remained essentially unchanged within the SGLT-2iG despite increased urinary excretion of these electrolytes; stable serum sodium despite natriuresis reflects compensatory RAAS activation and ECW adjustment [64], and the reported antikalemic effect of SGLT-2 inhibitors is particularly relevant in this population at high risk of hyperkalemia [65,66,67]. Regarding the impact of SGLT-2 inhibitors on fluid balance and body composition (assessed by BIA) and nutrition using NUS, in our study, within SGLT-2iG, BFM (25.2 to 23.1 kg, *p* = 0.02) and FFM (50.7 to 47.8 kg, *p* = 0.01) decreased, with stable BCM and improved PhA. This suggests that the body-weight loss in this group (75.7 to 70.8 kg; *p* = 0.001) was more likely driven by a reduction in body water than by a loss of muscle mass or malnutrition, a critical issue in CKD patients prone to sarcopenia [68], and this is consistent with the reported capacity of SGLT-2 inhibitors to shift substrate utilization from glycolysis towards fatty-acid and ketone-body oxidation [69]. Unlike traditional diuretics, SGLT-2 inhibitors may facilitate a selective reduction in adipose tissue and ECW, potentially aiding fluid-overload management without compromising cellular nutritional status [70]. Given the observational design, these body-composition findings are descriptive and cannot be attributed to SGLT-2 inhibition alone.

Contrary to traditional concerns about caloric deprivation secondary to sustained glucosuria, in our cohort, SGLT-2 inhibitor-associated weight and fat loss occurred without deterioration in muscle mass or strength, and NUS-derived muscle measures (*Y*-axis, CS-MARF, MARFI_h_) and HGS were preserved or slightly improved, contrasting with the sarcopenia frequently associated with both SGLT-2 inhibitors and maintenance HD [71]. However, these findings should not be ascribed to SGLT-2 inhibition. Throughout the study period, no structured intradialytic nutrition or exercise program was in place, and patients received standard medical care, including, in some instances, nutritional supplementation that was not systematically recorded. Consequently, the body-composition and muscle-mass results reported here should be regarded as hypothesis-generating and will require confirmation in adequately controlled studies.

Notwithstanding the theoretical apprehension surrounding the increased incidence of adverse events, specifically UTIs, genital mycotic infections, and EDKA in patients receiving KRT, current evidence suggests a favorable safety profile. Even in patients with preserved RKF, where glucosuric potential remains partially intact, emerging data from small-scale interventional trials and retrospective cohorts consistently demonstrate a tolerability profile congruent with our observations [11,24]. Crucially, our study reported no serious adverse events necessitating permanent treatment discontinuation, a finding that aligns with post hoc analyses of landmark clinical trials and case series in PD and iHD populations [18]. These results further corroborate recent evidence indicating that SGLT-2 inhibitors can be safely initiated in dialysis-dependent patients without evidence of significant drug accumulation or deleterious safety signals [18]. Nevertheless, large-scale, long-term RCTs remains imperative to definitively establish the long-term efficacy and safety of SGLT-2 inhibition in this high-risk population.

Several limitations should be noted. Firstly, and most importantly, treatment was allocated at the center level: all patients treated with SGLT-2 inhibitors were recruited from one hospital and all controls from the other, and the multiparametric dry-weight assessment (BIA and POCUS) was applied only at the study center. This introduces a structural, center-related bias that no cross-center analysis can fully eliminate. Several factors partially mitigate this concern: both centers followed an identical dialysis framework (the same IHDIP-based prescribing and escalating protocol, the same modality, and identical BIA and NUS instrumentation), differing only in SGLT-2 inhibitor exposure and volume monitoring; the fluid-removal burden (IDWG and UFR per session) was comparable between groups; and our main inference rests on within-group longitudinal change, in which the stabilization or attenuated decline of RKF in the study group contrasts with the near-universal decline of RKF after HD initiation reported even under rigorous standardized fluid protocols [9,48,49]. Residual confounding by center and by indication cannot, however, be excluded. Second, the sample size was small (n = 32), limiting generalizability; because proportions therefore correspond to few patients and may overstate apparent differences, absolute numbers are reported throughout, and no formal between-group comparison was performed. Moreover, the between-cohort comparison was unadjusted and thus vulnerable to confounding by indication, reflected by the higher baseline prevalence of T2DM, smoking, NVAF and history of cardiovascular disease, together with the lower baseline RKF at the start of KRT, in the SGLT-2iG. As these are all determinants of faster RKF decline, these imbalances would be expected to bias against rather than in favor of the treated group, making the observed RKF preservation unlikely to be an artifact of baseline differences. Given the observational design and the absence of multivariable adjustment, a direct renal effect of SGLT-2 inhibition cannot be fully separated from indirect effects mediated by cardiometabolic comorbidities, although the primary endpoint comprised direct measures of RKF (KrU, CrCl, 24 h UO), whose change is most plausibly explained by the intrarenal hemodynamic actions of this class, shown to be independent of diabetes. Third, the measurement RKF has inherent limitations in this setting: the KrU and CrCl obtained from timed urine collection are sensitive to volume status and collection completeness, and CrCl overestimates GFR, owing to tubular secretion, caveats that warrant a cautious, descriptive interpretation of the KrU and CrCl trajectories. Fourth, no structured nutritional or exercise program was in place, and routine real-world nutritional support was not systematically recorded; therefore, it cannot be excluded as a contributor to the observed body-composition and muscle findings. Likewise, the numerous secondary laboratory and body-composition variables were not governed by a pre-specified hypothesis, and are reported only as exploratory, descriptive observations. Finally, the 12-month follow-up, although not negligible, is relatively short and, together with the small sample, limits the characterization of long-term efficacy and safety, underscoring the need for larger trials with longer observation periods. The main strength of our study lies in its use of real-world data, the direct and serial quantification of RKF (monthly KrU and 24 h UO collections), and the consistency of the results across multiple clinical, biochemical and ultrasound parameters, including quantitative BIA, to directly assess fluid dynamics and body composition. These features, together with the hypothesis-generating nature of our findings, provide a sound rationale for the adequately powered randomized trials now needed to confirm them.

## 5. Conclusions

In conclusion, in this two-center, real-world cohort, the use of SGLT-2 inhibitors in incident iHD patients was associated with preservation of RKF and diuresis over the first year, a change that reached statistical significance only for 24 h UO, while the trends in KrU and CrCl did not, alongside a reassuring short-term safety profile with no deterioration in muscle mass or strength. Because treatment was assigned at the center level, these associations cannot be interpreted as a proven causal effect, and residual confounding cannot be excluded; the favorable body-composition findings cannot be causally attributed to SGLT-2 inhibition. Nevertheless, the within-group stabilization of KrU and UO contrasts with the expected post-HD decline of RKF and is biologically plausible. Given that the preservation of RKF is a critical determinant of cardiovascular stability, metabolic balance, and survival in the dialysis population, these hypothesis-generating findings should be interpreted with caution and provide a real-world rationale for the adequately powered RCTs now required to confirm whether SGLT-2 inhibition preserves RKF and prolongs iHD in this population.

## Figures and Tables

**Table 1:** Patients’ clinical characteristics according to group.

Variable	Overall n = 32	Non-SGLT-2iG n = 17	SGLT-2iG n = 15	*p*-Value
Demographic parameters				
Age (years), mean (SD)	69.9 (11.2)	66.3 (10.2)	73.9 (11.3)	0.05
Sex (Men), n (%)	22 (68.8)	11 (64.7)	11 (73.3)	0.6
Etiology of CKD, n (%)				0.45
DKD	9 (28.1)	4 (23.5)	5 (33.3)	
GD	3 (9.4)	1 (5.9)	2 (13.3)	
CTIN	2 (6.3)	1 (5.9)	1 (6.7)	
ADPKD	5 (15.6)	4 (23.5)	1 (6.7)	
DKD + GD	2 (6.3)	0 (0)	2 (13.3)	
DKD + NTIC	1 (3.2)	0 (0)	1 (6.7)	
CKDu	10 (31.1)	7 (41.2)	3 (20)	
HTN, n (%)	31 (96.9)	16 (94.1)	15 (100)	0.3
Loop diuretics, n (%)	32 (100)	17 (100)	15 (100)	1
ACEi/ARB, n (%)	31 (96.9)	16 (94.1)	15 (100)	0.3
T2DM, n (%)	18 (56.3)	7 (41.2)	11 (73.3)	0.03
Obesity, n (%)	12 (37.5)	6 (35.3)	6 (40)	0.78
Smoking, n (%)	20 (62.5)	8 (47.1)	12 (80)	0.001
Charlson index (points), mean (SD)	6.7 (2.0)	6.4 (1.5)	7.1 (2.5)	0.32
cardiovascular disease, n (%)	12 (37.5)	3 (17.7)	9 (60)	0.01
AMI, TIA	16 (50)	6 (35.3)	10 (66.7)	0.08
NVAF	8 (25)	1 (5.9)	7 (46.7)	0.008
Residual kidney function parameters at the start of KRT				
24 h UO (mL/24 h) mean (SD)	1967 (690.4)	2250 (754.2)	1646 (445.4)	0.009
KrU (mL/min/1.73 m^2^), mean (SD)	5.45 (1.5)	6 (1.5)	4.9 (1.3)	0.03
CrCl (mL/min/1.73 m^2^), mean (SD)	10.4 (3.5)	10.3 (3.3)	10.7 (3.9)	0.94
Hemodialysis parameters				
Baseline 1 session per week (%)	93.8	100	86.7	0.12
1WHD (%) at 12 months	62.5	46.7	80	0.06
Baseline Dry weight (kg), mean (SD)	76.9 (16.0)	78.0 (19.0)	75.7 (12.5)	0.69
Baseline Kt (L), mean (SD)	49.1 (6.0)	45.5 (1.4)	53.3 (6.5)	0.001
Baseline QB (mL/min), mean (SD)	347.8 (17.8)	346.4 (15.6)	349.5 (20.5)	0.63
Baseline APF (mL/min), mean (SD)	−179.7 (31.4)	−196.1 (10.9)	−161.1 (36.7)	0.001
Baseline VPF (mL/min), mean (SD)	175.0 (20.8)	190.6 (14.0)	157.2 (9.9)	0.000
Baseline SBP (mmHg), mean (SD)	150.0 (17.1)	143.7 (17.5)	157.1 (13.9)	0.025
Baseline DBP (mmHg), mean (SD)	77.4 (12.7)	73.3 (14.7)	82.1 (8.1)	0.049
Total HD sessions at the 12-month follow-up	1752	900	852	NA
IDWG (Kg) at the 12-month follow-up, mean (SD)	1.51 (1.32)	1.47 (1.30)	1.55 (1.35)	0.86
UFR per session (mL/kg/h) at the 12-month follow-up, mean (SD)	5.18 (4.12)	5.03 (3.84)	5.35 (4.42)	0.82
Arteriovenous fistula (%)	71.9	70.6	73.3	0.86
Scales of risk malnutrition baseline				
MIS (points)	6.3 (4.6)	6.8 (4.5)	5.8 (4.7)	0.6
Patients with MIS ≥ 8 points, n (%)	9 (28.1)	5 (29.4)	4 (26.7)	0.86
MST ≥ 2 points, n (%)	4 (12.5)	4 (23.5)	0 (0)	0.045
PEW (score 0–3), n (%)	21 (65.3)	12 (70.6)	9 (60)	0.5
No PEW (score 4), n (%)	11 (34.4)	5 (29.4)	6 (40)	
SARC-F ≥ 4 points, n (%)	5 (15.6)	4 (23.5)	1 (6.7)	0.19
Sarcopenia (EWGSOP2), n (%)	8 (25)	6 (35.3)	2 (13.3)	0.2
Dynapenia, n (%)	15 (46.9)	8 (47.1)	7 (46.7)	0.9

Abbreviations: NA: Not applicable/available; SD: standard deviation; %: percent; CKD: chronic kidney disease; DKD: diabetic kidney disease; GD: glomerular disease; CTIN: chronic tubulo-interstitial nephropathy; ADPKD: autosomal dominant polycystic kidney disease; CKDu: chronic kidney disease of unknown etiology; AMI: acute myocardial infarction; TIA: transient ischemic attack; NVAF: non-valvular atrial fibrillation; HTN: hypertension; ACEi/ARB: angiotensin-converting enzyme inhibitors/angiotensin II receptor blockers; KRT: kidney replacement therapy; KrU: residual kidney urea clearance urine 24 h; CrCl: residual kidney creatinine clearance urine 24 h; UO: urine output; 1WHD: once-weekly hemodialysis session; IDWG: interdialytic weight gain; Kt/V: dialysis adequacy index based on urea clearance; Kt: total urea clearance; nPCR: normalized protein catabolic rate; QB: blood flow rate; APF: arteriovenous pump flow; VPF: venous pump flow; SBP: systolic blood pressure; DBP: diastolic blood pressure; MIS: malnutrition inflammation score; MST: malnutrition screening tool; PEW: protein-energy wasting; SARC-F: sarcopenia-F; EWGSOP2: European Working Group on Sarcopenia in Older People.

**Table 2:** Primary outcome data according to RKF preservation over 1 year.

	24 h UO (mL/24 h), Mean (SD)		KrU (mL/min/1.73 m^2^), Mean (SD)		CrCl (mL/min/1.73 m^2^), Mean (SD)	
	Control	SGLT-2iG	Control	SGLT-2iG	Control	SGLT-2iG
Baseline (A)	2092.9 (739)	1620 (437.9)	5.8 (1.9)	4.7 (1.2)	9.9 (2.9)	11 (3.8)
At 3 Months	2041.2 (784.1)	1946.7 (475.3)	5.5 (1.9)	5.6 (1.3)	9.2 (2.9)	13.7 (4.5)
At 6 Months	2073.5 (723.5)	2100 (618.8)	5.7 (1.6)	6 (1.8)	10 (4.7)	14.6 (6.5)
At 9 Months	2135 (575.5)	2130 (599.3)	5.6 (1.9)	6.0 (2.1)	9.7 (4.6)	13.6 (8.2)
At 12 Months (B)	1890 (839.3)	2193.3 (642.8)	4.9 (2.1)	6.3 (2.6)	8 (4.3)	14.5 (10.8)
p (A–B)	0.34	<0.001	0.08	0.06	0.08	0.22

Data are shown as mean (SD: standard deviation). Control: iHD-non-SGLT-2iG; KrU: residual kidney urea clearance urine 24 h; CrCl: residual kidney creatinine clearance urine 24 h; UO: urine output. p (A–B) denotes the change from baseline (A) to 12 months (B): within-group (paired *t*-test) under the Control and SGLT-2iG columns. Baseline corresponds to the initiation of the SGLT-2 inhibitor (within the first three months of KRT; the equivalent time point was used for the control group).

**Table 3:** Safety outcomes after 12-month follow-up.

	Control	SGLT-2iG
N	17	15
Hypoglycemia, n (%)	3 (17.6)	0 (0)
CVE, n (%)	6 (35.3)	1 (6.7)
Limb amputation, n (%)	0 (0)	0 (0)
Hypersensitivity, n (%)	0 (0)	0 (0)
Death, n (%)	1 (5.9)	1 (6.7)
CVE, n (%)	0 (0)	1 (6.7)
Death (non-cardiovascular cause), n (%)	1 (5.9)	0 (0)
Transplant, n (%) *	3 (17.6)	2 (13.3)
Diabetic ketoacidosis, n (%)	0 (0)	0 (0)
Genital Infection, n (%)	0 (0)	0 (0)
Urinary tract infection, n (%)	0 (0)	0 (0)
COVID, n (%)	3 (17.6)	2 (13.3)
Catheter-related infection, n (%)	3 (17.6)	2 (13.3)
CVC-associated bacteremia	2 (11.8)	1 (6.7)
Infection ECS	1 (5.9)	1 (6.7)
Hospitalizations, n	6	5
Respiratory infections (including COVID), n	3	2
Bloodstream infection, n	2	1
Cholecystitis, n	0	1
Acute myocardial infarction, n	0	1
Acute heart failure, n	1	0
Number IDH episodes/total sessions (%)	16/900 (1.78)	10/852 (1.17)

%: Percentage; n: number; CVE: cardiovascular event; CVC: central venous catheter; ECS: external catheter site; IDH: intradialytic hypotension. * Transplants in both groups occurred one year after the end of the study follow-up period. Safety outcomes are reported descriptively as counts and percentages; given the small number of events, no formal between-group statistical testing was performed.

## Data Availability

No new data were created or analyzed in this study. The data used to support the findings of this study are available from the corresponding author upon request (contact J.C.D.L.F., jflomer@mde.es). We confirm that all the figures and tables are the original work of this manuscript’s authors. The authors of this manuscript have created it all; it has not been adapted from other authors, and the authors do not present an online link.

## Supplementary Materials

The following supporting information can be downloaded at https://www.mdpi.com/article/10.3390/medicina62101972/s1. Table S1: Evolution of serum and urinary analytical parameters in both groups after 12 months of follow-up; Table S2: Evolution of anthropometry variables, bioimpedance and nutritional ultrasonography parameters in both groups after 12 months of follow-up.
