# Supplementary material for: Effects of Sodium-Glucose Cotransporter Type 2 Inhibitors on Residual Kidney Function in Patients Undergoing Incremental Hemodialysis: A Two-Center, Retrospective, Exploratory Cohort Study
Source: Medicina (Kaunas). 2026 Oct 12;62(10):1972. doi: 10.3390/medicina62101972 (PMC13707212; doi:10.3390/medicina62101972)
Supplement: Supplementary file 1 [file medicina-62-01972-s001.zip › medicina-4512711-supplementary.pdf]

Table S1. Evolution of serum and urinary analytical parameters in both groups after 12 months of follow-up.

	Baseline (A)		6 months		12 months (B)		p Value (A-B)		Dif-p Value (C-D)
	Control	SGLT-2iG	Control	SGLT-2iG	Control	SGLT-2iG	Control (C)	SGLT-2iG (D)	
Cr serum (mg/dL), mean (SD)	6.6 (1.3)	5.7 (2.7)	7 (2.2)	5.4 (2.1)	7.5 (1.5)	5.9 (2.3)	<0.001	0.71	0.07
U serum (mg/dL), mean (SD)	170 (45.1)	163.9 (37.6)	187.6 (61.7)	152.3 (48.7)	156.6 (47.8)	152.5 (50.7)	0.15	0.41	0.95
Albumin (g/dL), mean (SD)	4 (0.4)	3.2 (0.3)	4 (0.3)	3.5 (0.3)	4 (0.4)	3.5 (0.2)	0.25	0.02	0.09
Gluc serum (mg/dl), mean (SD)	110.6 (37.4)	134.1 (37.2)	112.1 (35.7)	126.3 (38.6)	103.3 (34.1)	123.1 (26.6)	0.66	0.26	0.67
Hb A1c (g/dl), mean (SD)	5.9 (1)	6.3 (0.7)	5.5 (0.6)	6.1 (0.5)	5.8 (0.4)	6 (0.6)	0.46	<0.001	0.44
Hb (g/dL), mean (SD)	10.1 (1.2)	10.4 (1.4)	11 (1.3)	11.6 (0.8)	10.8 (0.8)	12.3 (0.8)	0.07	0.001	0.09
Fe (mg/dL), mean (SD)	50.9 (16.5)	66.5 (30)	74.1 (25.8)	78.9 (33.1)	71.2 (27.6)	68.7 (19.5)	0.03	0.75	0.13
TSAT (%), mean (SD)	21.6 (7.6)	25.7 (11.6)	32.4 (10)	34.8 (13.4)	29.4 (8.8)	34.1 (5.9)	0.02	0.05	0.77
Transferrin (mg/dL), mean (SD)	188.5 (38.2)	187.5 (27.4)	175.9 (24.6)	184.3 (33)	176.9 (36.8)	178.9 (36.6)	0.33	0.16	0.83
Ferritin (ng/mL), mean (SD)	446.2 (367.3)	528.3 (252.7)	809.2 (487.3)	680.5 (316.5)	711.1 (445.4)	676 (292.5)	0.007	0.01	0.38
LDL (mg/dL), mean (SD)	51.9 (25)	80.9 (38.9)	63.9 (25)	69.5 (28.1)	66.4 (25.6)	62.9 (24)	0.01	0.02	<0.007
HDL (mg/dL), mean (SD)	48.7 (21.4)	45.3 (10.5)	47.1 (14.6)	46.8 (9.7)	44.8 (13)	46.7 (10.2)	0.91	0.35	0.60
TG (mg/dL), mean (SD)	118.2 (42.2)	130.5 (72.7)	122.1 (48.3)	146.5 (88.2)	121.55 (49.4)	126.9 (58.4)	0.34	0.76	0.52
Cholesterol (mg/dL), mean (SD)	124.6 (29.7)	140.1 (38)	130 (28.7)	133.8 (33)	128.1 (29.9)	130.1 (26.7)	0.1	0.09	0.02
non-HDL-C (mg/dl), mean (SD)	75.9 (25)	95.8 (37.5)	82.9 (27.4)	87 (32.9)	83.3 (29.2)	83.4 (27.9)	0.09	0.03	0.005
B2M serum (pg/mL), mean (SD)	15.5 (1.7)	18 (2.7)	NA	NA	17.6 (3.1)	16.5 (2.3)	0.02	0.13	0.007
U.A. serum (mg/dL), mean (SD)	6.1 (1.6)	6.2 (1.8)	7.7 (6.4)	5.2 (1.5)	6.1 (1.2)	5.1 (1.2)	0.97	0.02	0.05
Na serum (mmol/L), mean (SD)	139 (3.3)	138.9 (2.1)	131 (3.7)	139.1 (2.9)	135.8 (2.3)	139.4 (3.1)	0.48	0.51	0.337
K^+ serum (mmol/L), mean (SD)	4.6 (0.6)	4.6 (0.8)	6.5 (6.6)	4.4 (0.5)	4.8 (0.6)	4.4 (0.4)	0.35	0.64	0.36
Cl^- serum (mmol/L), mean (SD)	100.5 (4)	104.4 (2.8)	98.8 (19.4)	104.9 (3.1)	101.1 (4)	104.8 (2.7)	0.85	0.73	0.9
Bicarbonate(mEq/L), mean (SD)	21.8 (2.8)	20 (2.3)	20.7 (3.1)	22.5 (1.4)	21.5 (2.4)	22.8 (1.3)	0.95	<0.001	0.013
Ca^{+2} serum (mg/dL), mean (SD)	9.1 (0.7)	8.6 (0.4)	9.1 (0.7)	8.8 (0.4)	9.2 (0.8)	8.8 (0.4)	0.5	0.21	0.735
Ionic Ca (mmol/L), mean (SD)	1.1 (0.1)	1.1 (0.1)	NA	NA	1.10 (0.11)	1.14 (0.07)	0.79	0.06	0.075
P serum (mg/dL), mean (SD)	5 (0.9)	4.8 (1)	5.1 (1.5)	4.9 (0.9)	5.4 (1.3)	4.8 (0.9)	0.25	0.98	0.392
Mg serum (mg/dL), mean (SD)	2.1 (0.2)	1.9 (0.2)	2.2 (0.3)	2.2 (0.1)	2.2 (0.3)	2.2 (0.3)	0.53	0.02	0.075
iPTH serum (pg/mL), mean (SD)	221.4 (115.5)	325,8 (185.2)	283.8 (170.1)	302.9 (219.7)	294.6 (204.4)	256.3 (220.3)	0.17	0.11	0.035
25OHD (ng/mL, mean (SD)	26.4 (10.5)	26.7 (7.4)	28.4 (11.8)	31.3 (3.6)	29.9 (16.7)	31.7 (4.4)	0.45	0.07	0.746
Residual diuresis (mL/24 h), mean (SD)	2092.9 (739)	1620 (437.9)	2073.5 (723.5)	2100 (618.8)	1890 (839.3)	2193.3 (642.8)	0.34	<0.001	0.007
Glucosuria (mg/dl), mean (SD)	2.9 (12.1)	14.2 (32)	1.5 (4.4)	317.3 (153.4)	1 (3.9)	314.3 (100.2)	0.33	<0.001	<0.001
U.A. urine (mg/24 h), mean (SD)	128.6 (67.4)	104 (50.9)	144 (77)	216.2 (95.5)	123.2 (71.9)	226.3 (148.9)	0.88	<0.001	0.06
U.A. Clr. urine 24h (ml/min), mean (SD)	1.3 (0.6)	1 (0.4)	4.2 (8.1)	1.5 (0.5)	1.3 (1)	1.7 (0.7)	0.7	<0.001	0.08

Na^+ urine 24h (mmol/24h), mean (SD)	132.5 (91.2)	74.8 (37.1)	164.1 (89.8)	165.4 (78)	161.3 (79.7)	174.2 (81.1)	0.28	<0.001	0.06
Na^+ urine single void (mmol/L), mean (SD)	62.8 (28)	40.7 (13)	68.2 (26.7)	79.4 (16.9)	56.7 (26.7)	82 (15.4)	0.83	<0.001	0.001
K^+ urine 24h (mmol/24h), mean (SD)	49.8 (17.2)	31.3 (13.9)	48.7 (19.6)	48.7 (13.6)	43.2 (18.8)	52.5 (16.4)	0.13	<0.001	<0.001
K^+ urine single void (mmol/L), mean (SD)	27.7 (9)	14.7 (8)	23.4 (7.6)	31.7 (11.6)	24.1 (8.1)	32.2 (7.7)	0.05	<0.001	<0.001
Cl^- urine 24h (mmol/24h), mean (SD)	57.4 (21.7)	123.8 (45.8)	54.5 (17.7)	106.4 (39)	53.9 (15.6)	112.7 (35.2)	0.55	0.36	0.350
Cl^- urine single void (mmol/L), mean (SD)	40.6 (11.9)	33.5 (14.3)	42.1 (10.3)	57.1 (13.5)	40.9 (9.8)	60.5 (10.6)	0.81	<0.001	<0.001
Ca^{+2} urine 24h (mg/24h), mean (SD)	33.4 (19.2)	28.6 (20.7)	32.3 (17.6)	65.2 (46.1)	32.7 (17.3)	63.4 (40.7)	0.55	<0.001	0.001
E.I. Ca^{+2} urine 24h, mean (SD)	0.6 (1.5)	0.2 (0.2)	0.6 (1.5)	0.4 (0.3)	0.7 (1.6)	0.4 (0.4)	0.16	0.01	0.006
Ca^{+2} Clr. urine 24h (ml/min), mean (SD)	0.3 (0.2)	0.3 (0.2)	0.2 (0.2)	0.4 (0.25)	0.2 (0.2)	0.6 (0.7)	0.08	0.04	0.017
P urine (mg/24h), mean (SD)	409.4 (137.2)	373.4 (193)	443 (143.9)	328.7 (124.7)	298.5 (180)	310.3 (120.8)	0.04	0.13	0.457
P Cl. urine 24h (ml/min), mean (SD)	5.5 (1.7)	6.3 (3.3)	6 (1.8)	4.8 (2.4)	5 (2.1)	5.3 (2.8)	0.43	0.3	0.715
T.R.P. urine 24h (%), mean (SD)	61.3 (23.8)	57.2 (16.7)	45 (23.7)	63.1 (16.9)	48.2 (24.2)	59.9 (19.1)	0.47	0.46	0.348
TMP/GFR Urine 24h, mean (SD)	2.3 (1)	2.4 (1)	1.8 (1.5)	2.4 (1)	2.1 (1.9)	2.5 (0.8)	0.21	0.54	0.208
uACR (mg/g), mean (SD)	1435.6 (983.6)	1965.5 (1726.4)	1734.5 (1890.1)	1322.5 (1111.9)	1838.7 (1984)	1068.2 (940.9)	0.32	0.02	0.016
uPCR (mg/g), mean (SD)	1888.9 (1264.3)	2319.3 (1934.8)	2294.1 (2242.7)	1647.3 (1315.4)	2425.3 (2338.1)	1168.9 (1117.5)	0.22	0.01	0.005
Proteinuria (g/24h), mean (SD)	1.9 (1.3)	2.5 (2.1)	2.3 (2.4)	1.5 (1.2)	2.5 (2.5)	1.2 (0.9)	0.28	0.01	0.008

Data are shown as mean (SD: standard deviation) or percentage (%), NA: Not Applicable/Available. SGLT-2iG (-): Non-SGLT-2iG; Cr: creatinine; U: urea; Gluc: glucose; Hb: Hemoglobin; Fe: iron; TSAT: transferrin saturation; HDL: high-density lipoprotein; LDL: low-density lipoprotein; non-HDL-C: Non-HDL Cholesterol; TG: Triglycerides; B2M: Beta-2 microglobulin; U.A.: Uric Acid; Cl.: clearance; h: hours; Na^+: sodium; K^+: potassium, Cl^-: Chloride; Ca^{+2}: calcium; E.I.: excretion index; P: phosphorus; T.R.P.: Tubular Reabsorption of Phosphate; TMP/GFR: Tubular Maximum Reabsorption of Phosphate per Glomerular Filtratation Rate; Mg: magnesium; iPTH: intact parathyroid hormone; 25OHD: 25-hydroxyvitamin D; uPCR: Urine Protein/Creatinine Ratio; uACR: Urine Albumine/Creatinine Ratio.

Table S2. Evolution of anthropometry variables, bioimpedance and Nutritional ultrasonography parameters in both groups after 12 months of follow-up.

	**Baseline**		**At 6 months**		**At 12 months**		p value (Dif Baseline- at 12m)	
	**Control**	**SGLT-2iG**	**Control**	**SGLT-2iG**	**Control**	**SGLT-2iG**	**Control**	**SGLT-2iG**
Anthropometry variables								
Handgrip strength (kg), mean (SD)	20.06 (8.81)	20.03 (8.9)	NA	NA	19.9 (8.6)	23.1 (8.1)	0.62	<0.00
Triceps skinfold (mL), mean (SD)	15.1 ± 7.9	14.0 ± 5.9	NA	NA	15.3 ± 7.6	13.8 ± 5.0	0.26	0.7
Suprailiac skinfold (mL), mean (SD)	21.1 ± 10.4	18.1 ± 8.5	NA	NA	20.8 ± 10.0	17.1 ± 7.6	0.17	0.09
Waist circumference (cm), mean (SD)	31.3 ± 4.8	29.5 ± 3.4	NA	NA	31.7 ± 4.8	28.3 ± 3.7	0.24	0.003
Arm circumference (cm), mean (SD)	85.2 ± 17.6	83.5 ± 14.1	NA	NA	85.2 ± 17.1	82.4 ± 13.8	0.72	0.04
SBP (mmHg), mean (SD	141.6 ± 17.5	157.1 ± 13.9	143.7 (12.6)	132.3 (7.7)	142.2 ± 14.2	132.1 ± 9.7	0.91	<0.001
DBP (mmHg), mean (SD	71.1 ± 14.3	82.1 ± 8.1	77.8 (11.5)	75.5 (6.7)	73.9 ± 9.3	76.8 ± 6.0	0.48	0.08
Weight (kg), mean (SD)	78 (19)	75.7 (12.5)	77.5 (19.2)	72.6 (11.6)	79.1 (19.5)	70.8 (11.3)	0.21	<0.001
BMI (kg/m^2), mean (SD)	29.2 (6.1)	27.8 (3.3)	29 (6.2)	26.6 (2.9)	29.3 (6.2)	26 (2.9)	0.21	<0.001
Bioimpedance parameters								
ECW/TBW ratio, mean (SD	0.4 (0)	0.4 (0)	0.4 (0)	0.4 (0)	0.4 (0)	0.4 (0.1)	0.95	0.72
TBW (L), mean (SD)	37.9 (6.8)	38.3 (8.7)	38 (7.4)	35.7 (6.3)	38.7 (7.5)	34.8 (6.4)	0.85	0.02
ICW (L), mean (SD)	22.5 (4.1)	22.7 (4.6)	22.5 (4.1)	21.8 (3.9)	22.9 (4.2)	21 (3.7)	0.33	0.02
ECW (L), mean (SD)	15.4 (3)	15.6 (4.1)	15.5 (3.6)	13.9 (2.5)	15.8 (3.7)	14.5 (3.7)	0.87	0.31
ASMI (kg/m2), mean (SD)	7.3 (1.1)	8.1 (1.5)	7.3 (1.1)	8.2 (1.5)	7 (1)	8 (1.2)	0.006	0.58
BFM (kg), mean (SD)	25.3 (6.4)	25.2 (8.7)	25.1 (6.5)	23.8 (8.3)	25.7 (6.6)	23.1 (8.2)	0.21	0.02
LBM (kg), mean (SD)	54.6 (13.3)	46 (8.2)	54.2 (13.4)	45.5 (7.6)	55.4 (13.7)	45.3 (7.4)	0.21	0.47
FFM (kg), mean (SD)	52.7 (12.6)	50.7 (9.8)	52.4 (12.7)	48.9 (8.7)	53.5 (13)	47.8 (8.2)	0.21	0.01
BCM (kg), mean (SD)	34.2 (1.6)	31.5 (5.6)	34.2 (1.6)	31.4 (5.4)	32.1 (8.2)	31.3 (5.7)	0.3	0.68
Visceral fat area (cm^2), mean (SD)	117 (46.1)	113.2 (47.9)	117 (46.1)	111.4 (53.5)	110.0 (44.5)	101.94 (43.9)	0.61	0.39
Phase angle (50hz), mean (SD)	5.3 (0.9)	5 (1)	5.3 (0.9)	5.1 (1)	5.4 (0.5)	5.2 (0.9)	0.11	0.006
BMTR (kCals/24 h), mean (SD)	1425 (56.7)	2327.1 (3507.1)	1425 (56.7)	1418 (175.6)	1429.3 (59)	1405.4 (173.6)	0.33	0.32
Nutritional ultrasonography parameters								
Y-axis (mm), mean (SD)	9.5 ± 1.0	9.3 ± 2.2	NA	NA	9.3 ± 0.7	10.0 ± 1.9	0.22	<0.001
Y-axis/height (mm/m^2), mean (SD)	3.5 ±0.7	3.5 ±0.6	NA	NA	3.5 ±0.6	3.7 ±0.8	0.22	<0.001
X-axis (mm), mean (SD)	32.4 (5.1)	31.4 (4.5)	NA	NA	31.7 ± 4.9	32.4 ± 4.3	0.6	0.53
CS-MARF (cm^2), mean (SD)	3.1 ± 0.4	2.6 ± 0.9	NA	NA	2.9 ± 0.8	3.1 ± 0.4	0.06	0.003
MARFIh (cm^2/m^2), mean (SD)	1.2 ± 0.2	1.0 ± 0.3	NA	NA	1.1 ± 0.3	1.2 ± 0.2	0.07	0.003
SMF (mm), mean (SD)	7.8 ± 2.8	8.6 ± 2.9	NA	NA	8.5 ± 2.9	7.9 ± 2.7	0.37	0.19
Transverse PPVF (cm), mean (SD)	0.8 ± 0.3	0.6 ± 0.3	NA	NA	0.8 ± 0.3	0.5 ± 0.2	0.66	0.003
Transverse SSCF (cm), mean (SD)	0.9 ± 0.3	0.8 ± 0.3	NA	NA	0.9 ± 0.3	0.7 ± 0.2	0.36	0.04
Transverse DSCF (cm), mean (SD)	1.3 ± 0.5	1.2 ± 0.4	NA	NA	1.3 ± 0.5	1.1 ± 0.4	0.41	0.04

Data are shown as means (SD: standard deviation), m: months; SBP: systolic blood pressure; DBP: diastolic blood pressure; BMI: body mass index; TBW: total body water; ECW: extracellular water; ICW: intracellular

water; ASMI: appendicular skeletal muscle mass index; BFM: body fat mass; LBM: lean body mass; FFM: fat-free mass; BCM: body cell mass; BMTR: basal metabolic rate; CS-MARF: cross-sectional muscle area of the rectus femoris; MARFIh: muscle area of the rectus femoris index adjusted to height; SMF: supramuscular fat; PPVF: preperitoneal visceral fat; SSCF: superficial subcutaneous fat; DSCF: deep subcutaneous fat.
